# Glucocorticoid use and skin cancers

**DOI:** 10.1038/sj.bjc.6601221

**Published:** 2003-08-26

**Authors:** M P Purdue

**Affiliations:** 1 Division of Preventive Oncology, Cancer Care Ontario, Toronto, Canada M5G 2L7

**Sir**,

Karagas *et al* (2001) reported modest elevations in risk of basal cell carcinoma (BCC) and squamous cell carcinoma (SCC) accompanying oral steroid use but not for use of inhaled steroids. The absence of effect from inhalants was attributed to their limited systemic effects of glucocorticoids. Another possible explanation, however, is the presence of bias owing to the confounding effects of asthma, a condition for which inhaled glucocorticoids are often used.

Asthma and other atopic conditions such as eczema and hay fever are often treated with corticosteroids and have been found by some epidemiologic studies to be associated with a reduced risk of many types of cancers, although these findings are not consistent l (Vena *et al*, 1985; McWhorter, 1988; Mills *et al*, 1992; Kallen *et al*, 1993; Vesterinen *et al*, 1993). It has been postulated that the hypersensitivity to allergens associated with these conditions protects against cancer by providing superior recognition of, and immune response to, tumour antigens (Volkers, 1999). Consequently, if atopic conditions do confer protection from cancer, the failure to adjust for atopic disease in the original analysis would bias the estimated relative risks of both oral and inhaled glucocorticoids towards the null.

A reanalysis of data pertaining to oral glucocorticoid use presented in the paper by Karagas *et al* suggests the possibility of such confounding. Table 1

**Table 1:** Distribution of oral glucocorticoid use and skin cancer (based on data presented in Table 1 of Karagas *et al*, 2001)

		**Controls**	**BCC**	**SCC**
**Steroid use^a^**	**Reason**	***N* (%)**	***N* (%)**	***N* (%)**
No		491 (88.8)	534 (85.9)	245 (81.9)
Yes	Any	31 (5.6)	44 (7.1)	27 (9.0)
	Treat possible atopic conditions^b^	16 (2.9)	14 (2.3)	10 (3.3)
	Treat possible non-atopic conditions^c^	15 (2.7)	30 (4.8)	17 (5.7)

BCC=basal cell carcinoma; SCC=squamous cell carcinoma.

a Use of oral glucocorticoid use for 1 month or longer.

b Includes ‘respiratory conditions and asthma’ and ‘allergies’.

c Includes ‘musculoskeletal and connective tissue disease’, ‘neoplasm’, ‘gastrointestinal disease’, ‘other’.

provides the distribution among cases and controls of oral glucocorticoid use (use for 1 month or longer) subcategorised by reason for possible steroid use. The data for steroid users were pooled into two groups: one group representing subjects treating possibly atopic conditions (specifically, those using steroids for ‘respiratory conditions and asthma’ and ‘allergy’) and the other group representing subjects with other conditions (those categorised under other reasons).

Crude odds ratios with accompanying 95% confidence intervals were calculated to measure the association between risk of BCC and SCC and oral steroid use (Table 2Table 2

**Table 2:** Association between oral glucocorticoid use to treat possible atopic/non-atopic conditions and skin cancer risk

		**BCC**	**SCC**
**Steroid use^a^**	**Reason**	**OR (95% CI)**	**OR (95% CI)**
No		—	—
Yes	Any	1.31 (0.81–2.10)	1.75 (1.02–2.99)
	Treat possible atopic conditions^b^	0.80 (0.39–1.67)	1.25 (0.56–2.80)
	Treat possible non-atopic conditions^c^	1.84 (0.98–3.46)	2.27 (1.12–4.62)

equals;basal cell carcinoma; SCC=squamous cell carcinoma; OR=odds ratio; CI=confidence interval.

a Use of oral glucocorticoid use for one month or longer.

b Includes ‘respiratory conditions and asthma’ and ‘allergies’.

c Includes ‘musculoskeletal and connective tissue disease’, neoplasm, gastrointestinal disease, other.

). Stronger increases in risk of each skin cancer were found for steroid use to treat (supposedly) non-atopic conditions, compared to nonusers. Steroid use to possibly treat atopic conditions was not found to be associated with either type of cancer. These findings suggest that the effects of atopic conditions may confound the association between glucocorticoids and skin cancer risk. The analysis is limited in that the odds ratios have not been adjusted for other confounding factors, although the adjusted odds ratios in the original analysis were very similar in magnitude to the unadjusted odds ratios. Another limitation involves the fact that four cases (two BCC, two SCC) that received steroid treatment for conditions in more than one category were not identified as such in the original table, and so could not be adjusted for in this analysis. However, other analyses involving different assumptions about the possible joint conditions suggested that the findings would not be greatly influenced by this problem. Both of these issues could be addressed by the authors through reanalysis of the original study data. It would also be interesting to know whether a similar reanalysis of inhaled steroid use resulted in comparable findings.
